# Patients' and GPs' expectations regarding healthcare-seeking behaviour: a Norwegian comparative study

**DOI:** 10.3399/bjgpopen18X101615

**Published:** 2018-11-14

**Authors:** Torunn Bjerve Eide, Jørund Straand, Elin Olaug Rosvold

**Affiliations:** 1 PhD Student, Department of General Practice, Institute of Health and Society, University of Oslo, Oslo, Norway; 2 Leader, Department of General Practice, General Practice Research Unit (AFE), Institute of Health and Society, University of Oslo, Oslo, Norway; 3 Professor, Department Leader, Department of General Practice, Institute of Health and Society, University of Oslo, Oslo, Norway; 4 Professor, Leader of the National Research School in General Practice, Department of General Practice, Institute of Health and Society, University of Oslo, Oslo, Norway

## Abstract

**Background:**

GPs are Norwegian patients' first contact point with the healthcare system for most medical problems. However, little is known regarding GPs' expectations towards their patients' healthcare-seeking behaviour, or whether doctors and patients have coinciding expectations of what GPs can do for their patients.

**Aim:**

To investigate patients' and GPs’ expectations regarding patients’ healthcare-seeking behaviour in primary care, and to make comparisons between the two.

**Design & setting:**

Norwegian data from the Quality and Costs of Primary Care in Europe (QUALICOPC) questionnaire study, with information from GPs and their patients.

**Method:**

Binary logistic regression was used to investigate associations between expectations, sex and age of GPs and patients, list size, and geographical location of practice. Results are presented as odds ratios (ORs) with 95% confidence intervals (CIs). Expectation differences between GPs and patients were analysed using generalised estimating equations (GEEs). Due to multiple testing, Bonferroni correction was used to define significance level at *P*≤0.002.

**Results:**

In total, 198 GPs (39.1% female) and 1529 patients (61.9% female) responded. No associations with sex or age were found for the GPs' expectations regarding patients' healthcare-seeking behaviour. Among patients, fewer males than females expected that most people would see their GP for sprained ankle (OR 0.7, 95% CI = 0.5 to 0.9), finger cut (OR 0.6, 95% CI = 0.4 to 0.7), smoking cessation (OR 0.6, 95% CI = 0.5 to 0.8), or anxiety (OR 0.4, 95% CI = 0.3 to 0.6). Older patients (aged >65 years) found it more important than younger patients to see a doctor in the presence of medical symptoms. GPs had higher expectations than their patients that people in general would see them for deteriorated vision (OR 4.2, 95% CI = 2.5 to 6.9), sexual problems (OR 1.8, 95% CI =1.3 to 2.6), and anxiety (OR 3.0, 95% CI =1.5 to 6.0).

**Conclusion:**

For several common health problems, males are less likely than females to believe that people will see their GP. GPs may overestimate to what degree their patients will see them for a number of common medical problems.

## How this fits in

Norway has a strong primary health care system, and GPs offer comprehensive medical services to their patients. However, little is known about whether GPs and their patients have similar expectations regarding which medical issues that will bring people to see their GP. This study found that GPs may overestimate to what degree their patients will see them for common medical problems, in particular for psychosocial issues. Patients' sex and age affect their healthcare-seeking behaviour.

## Background

Healthcare systems with a strong primary care sector are associated with better population health,^1^ lower rates of avoidable hospitalisation^1^ and a better patient perception of primary care quality.^2^ Health systems with strong primary care have better cost effectiveness and slower growth in health expenditures.^3^

In 2001, Norway introduced the regular general practitioner (RGP) scheme, assigning every inhabitant to an individual GP. In 2016, 70% of Norwegians had one or more visits to their RGP, with a mean of 2.6 visits per inhabitant.^4^ GPs are patients' first contact point with the health services for most medical problems, and offer a comprehensive range of services.^5,6^ GPs also have a gatekeeping role for access to specialised healthcare services. Most citizens therefore have some knowledge about their RGP and the medical services they offer.

With this in mind, it is of interest to know what kind of medical help patients expect to receive in general practice, and whether doctors and patients have coinciding expectations of what GPs can do for their patients. Extensive research exists on what kind of symptoms and complaints bring people to the GP.^4,6–12^ While most studies focus on the symptoms and medical issues addressed in the consultation, less is known regarding patients' preconceived beliefs about what kind of medical problems a GP can help with.^13,14^ Most people have a notion regarding which conditions they can safely handle themselves; thus, most minor complaints will not lead to a visit to their GP.^15^ When someone consults a GP, they probably have an expectation that this will somehow help or benefit them. However, information is lacking about such expectations. Patients’ experiences with the healthcare system may also influence their propensity to seek health care. In a multinational European study, it was found that patients who reported good access and continuity, as well as good communication with their GP, had a higher propensity to seek care, especially for minor complaints.^16^ Among 23 GPs and their patients in Switzerland, Sebo *et al* found that GPs tend to underestimate patients' satisfaction while overestimating their expectations regarding structural aspects, such as access to care and presence of laboratory equipment, but the authors did not investigate expectations towards clinical problems.^17^ It is likely that GPs have expectations concerning which complaints and symptoms bring their patients to them. However, the present authors did not find studies on this issue, nor on whether or not GPs' and their patients' expectations coincide.

This study from Norwegian general practice aims to investigate patients' and GPs’ expectations concerning patients’ healthcare-seeking behaviour, and whether they are associated with GPs' or patients' sex or age, GP list size, or geographical location of the practices. Comparisons will be made between the patients' healthcare-seeking behaviour and the GPs' expectations.

## Method

This study uses data originating from the QUALICOPC study.^18^ A set of questionnaires for GPs and patients was developed by the QUALICOPC Partner Consortium, led by the Netherlands Institute for Health Services Research (NIVEL). Across Europe, participating GPs completed a questionnaire reporting information about their practices. Questionnaires were distributed to patients in GP waiting rooms on one day (randomly selected), and all participating patients had an appointment with the GP that same day; some questions related to that specific visit, and some were more general (see Box 1 for the phrasing of questions used in this study).

The questionnaires were derived from existing, validated questionnaires in three consensus rounds followed by a pilot study before a final revision. Translation to Norwegian was done using a 'forth and back' translation procedure, as described by Schafer et al.^19^ The questionnaires are described in further detail elsewhere,^19^ as is the implementation of the QUALICOPC study.^18^

### Sample

GPs in Norway were recruited through convenience sampling within formal and informal GP networks. Patients aged ≥18 years were approached by a field worker in the GP's waiting room before a consultation to request participation. The patient questionnaire was answered partly before and partly after the consultation. All questionnaires were answered anonymously. A unique identification number linked GP responses to the responses of their patients. Data collection took place from November 2012–April 2013.

### Main outcome measures

Box 1 shows all dependent variables from the GP and patient questionnaires that were included in the analysis. The GPs were asked to what extent they believed that their patients would contact them given a selection of health problems or symptoms. For each problem or symptom, the GPs were given four possible answers: (Almost) always (1); Usually (2); Occasionally (3); and Seldom/never (4). During analysis, answers were dichotomised: (1 + 2) and (3 + 4).

**Box 1.** Items included in the analysis from GP questionnaires and patient questionnaires. Questions from the Quality and Costs of Primary Care in Europe (QUALICOPC) study, 2012–2013

**Questions from the GP questionnaire**	
**In case of the following health problems, to what extent will patients in your practice population contact you as the first doctor?^a ^**(Only first contact, not for further diagnosis or treatment) Possible answers: (Almost) always, Usually, Occasionally, Seldom/Never	
Somatic problems	Child with severe cough^b^
	Man aged 24 with stomach pain^b^
	Woman aged 60 with deteriorating vision^b^
	Man aged 35 with sprained ankle^b^
	Woman aged 60 with polyuria
	Woman aged 60 with acute symptoms of paralysis/paresis
	Man aged 70 with joint pains
	Woman aged 75 with moderate memory problems
	Child aged 8 with hearing problem
	Man aged 28 with a first convulsion
	Man aged 45 with chest pain
	Woman aged 50 with a lump in her breast
	Woman aged 18 asking for oral contraception
Psychosocial problems	Man aged 32 with sexual problems^b^
	Physically abused child aged 13
	Anxious man aged 45^b^
	Couple with relationship problems^b^
	Woman aged 50 with psychosocial problems
	Man aged 52 with alcohol addiction problems
**Questions from the patient questionnaire**	
**Would most patients visit their GP for the following conditions? **Possible answers: Yes, Probably yes, Probably not, No, Don’t know	
Somatic problems	Child with severe cough^b^
	Stomach pain^b^
	Deteriorated vision^b^
	Sprained ankle^b^
	Cut finger that needs to be stitched
	Removal of a wart
	Blood in stool
	Help to quit smoking
Psychosocial problems	Sexual problems^b^
	Domestic violence
	Anxiety^b^
	Relationship problems^b^
Other	Routine health cheques
	Advice for choosing the best hospital/specialist
**How important would it be for you to see a doctor if you had…**Possible answers: Extremely important, Rather important, Somewhat important, Not important	
Somatic problems	Weight loss >2 kg in one month
	Shortness of breath with light exercise
	Chest pain when exercising
	Headache >1 day
	Abdominal pains >1 day
	Loss of consciousness/fainting
Psychosocial	Severe worries >1 month
**Do you expect to benefit from a visit to your GP for...**Possible answers: Yes, No, Don’t know	
Somatic problems	Stomach problems
	Diarrhoea
	Shoulder/neck pain
	Headache
	Flu
	Sore throat
	Feeling nauseous
	Feeling tired
Psychosocial	Feeling nervous
**Do you agree with the following statements?**Possible answers: Strongly agree, Agree, Disagree, Strongly disagree	
	In general, doctors can be trusted
	In general, people can be trusted

^a^In the English version of the questionnaire, the term 'first healthcare provider' was used, but in the Norwegian version this was translated to 'first doctor'.

^b^Included in regression analysis to compare responses from GPs and patients.

The patients were asked whether they believed that most patients would see their GP for a predefined selection of health problems, with five possible answers: Yes (1); Probably yes (2); Probably no (3); No (4); and Do not know (recorded as 'missing'). During analysis, answers were dichotomised to either Yes (1 + 2) or No (3 + 4). Patients were also asked if they expected to benefit from visiting their GP for the listed health problems, with the response alternatives Yes; No; and Do not know (recoded as 'missing'). Finally, the patients were asked how important it would be for them to see a doctor when experiencing the listed symptoms, with four possible answers: Extremely important (1); Rather important (2); Somewhat important (3); Not important (4). During analysis, they were merged into Important (1 + 2) or Not important (3 + 4).

For the participating GPs, sex, age, size of patient list, and urban or rural practice setting were recorded. For participating patients, sex and age were recorded.

### Statistics

A binary logistic regression model was used to analyse patients’ and GPs’ responses by their sex, age, and practice location, and, for GPs, by their patient list size.

To explore possible differences in patients' and doctors' expectations, seven comparable items were identified from the GP and patient questionnaires (Box 1). Due to the clustered structure of the material, with patients nested within GPs, a GEE logistic regression model was used, correcting for patients' and GPs' sex and age, and also practice location and the size of patient list of the GP that the patient had visited.

To correct for multiple testing, a Bonferroni correction was conducted based on the maximum number of tests^19^ for one questionnaire item. After calculating α = 0.05/19 = 0026, significance level was set at *P*≤0.002. Results with *P*<0.05 are also highlighted in the tables. ORs and percentages are given with 95% CIs. Analyses were performed in IBM SPSS Statistics (version 22).

## Results

Characteristics of the participating 198 GPs (39.1% female) and 1529 patients (61.9% female) are presented in Table 1.

**Table 1:** Demographics of patients (*n* = 1529) and GPs (*n* = 198) participating in the Norwegian part of the Quality and Costs of Primary Care in Europe (QUALICOPC) study, 2012–2013.

	**Total *n* (%)**	**Female *n* (%)**	**Male *n* (%)**
**Patients**			
**Total**	1529 (100.0)	916 (61.9)^a^	564 (38.1)^a^
**Age^b^**			
Range	18–93	18–91	18–93
Mean	48.7	46.2	52.5
**GPs**			
**Total**	198 (100.0)	77 (39.1)^c^	120 (60.9)^c^
**Age**			
Range	28–69	28–68	28–69
Mean	45.7	43.4	47.0
**Practice location** ^d^			
Large inner city	66 (33.8)	29 (38.7)	36 (30.3)
Suburbs	27 (13.8)	12 (16.0)	15 (12.6)
Small town	44 (22.6)	14 (18.7)	30 (25.2)
Mixed urban–rural	31 (15.9)	7 ( 9.3)	24 (20.2)
Rural	27 (13.8)	13 (17.3)	14 (11.8)
**Size of patient list** ^c^			
Range	250–1800	400–1500	250–1800
Mean	1093.4	1048.9	1122.6

^a^Missing data = 49. ^b^Missing data = 59. ^c^Missing data = 1. ^d^Missing data = 3.

Table 2 shows the GPs' answers to which health problems they believe would bring their patients to see them. Almost all GPs believed that patients would see them for common health problems such as severe cough, stomach pain, lump in breast, polyuria, joint pain, or anxiety. They less frequently expected patients to consult for convulsions, abuse, relationship problems, or alcohol problems. There were no significant (*P*≤0.002) associations with sex, age, list size, or location of practice, apart from lower expectation among urban GPs to be visited for a convulsion episode.

**Table 2.** 'In case of the following health problems, to what extent will patients in your practice population contact you as the first doctor?' Responses from GPs (*n* = 198) who participated in the Norwegian part of the Quality and Costs of Primary Care in Europe (QUALICOPC) study, 2012–﻿2013. Results given as valid percentages and ORs with 95% CIs, indicating the probability for the answer Always/Usually, with Occasionally/Never as reference by sex, age, list size, and practice location

Patient cases(age, years)	*n* ^a^	%(95% CI)	GP sex(reference: female)	GP age(reference: 36–59)		List size(reference: 901–1300)		Location of practice(reference: rural)
			**Male**	**≤35**	**≥60**	**≤900**	**>1300**	**Urban**
Child severe cough^b^	194	98.5 (96.4 to 100.0)	—^i^	—^i^	0.1 (0.0 to 0.8)^j^	0.3 (0.0 to 5.4)	0.5 (0.0 to 12.1)	0.8 (0.1 to 14.9)
Man (24) Stomach pain^c^	189	96.9 (94.4 to 99.0)	0.9 (0.1 to 5.1)	—^i^	0.7 (0.1 to 7.0)	0.8 (0.1 to 5.6)	1.2 (0.1 to 13.2)	2.2 (0.4 to 13.2)
Woman (60) Deteriorated vision^c^	175	89.7 (85.6 to 93.8)	1.5 (0.6 to 3.9)	1.1 (0.3 to 4.1)	0.9 (0.2 to 4.6)	0.9 (0.3 to 3.1)	0.6 (0.2 to 1.9)	0.9 (0.3 to 2.8)
Man (35) Sprained ankle^d^	146	75.3 (69.1 to 81.4)	0.9 (0.4 to 1.7)	0.8 (0.3 to 2.0)	1.1 (0.3 to 3.6)	0.6 (0.3 to 1.5)	0.9 (0.4 to 2.1)	0.3 (0.1 to 0.9)^j^
Woman (60) Polyuria^c^	187	95.9 (92.8 to 98.5)	0.9 (0.2 to 4.2)	0.5 (0.1 to 3.1)	0.6 (0.1 to 5.8)	0.9 (0.1 to 5.7)	1.1 (0.2 to 6.3)	0.3 (0.0 to 2.9)
Woman (60) Acute paresis^c^	138	70.8 (64.1 to 76.9)	0.7 (0.3 to 1.3)	1.2 (0.5 to 3.2)	0.9 (0.3 to 2.7)	1.8 (0.8 to 4.4)	2.9 (1.2 to 7.0)^j^	0.2 (0.1 to 0.5)^k^
Man (70) Joint pain^e^	188	97.4 (94.8 to 99.5)	0.5 (0.1 to 5.1)	0.7 (0.1 to 7.4)	0.4 (0.0 to 4.0)	0.3 (0.0 to 3.9)	0.2 (0.0 to 2.6)	1.8 (0.2 to 16.1)
Woman (75) Memory problems^d^	180	92.8 (88.7 to 96.4)	0.7 (0.2 to 2.4)	0.3 (0.1 to 1.3)	0.3 (0.1 to 1.3)	1.0 (0.3 to 4.1)	1.1 (0.3 to 5.0)	0.8 (0.2 to 3.2)
Child (8) Hearing problems^f^	172	87.8 (82.7 to 92.3)	1.3 (0.5 to 3.4)	1.0 (0.3 to 3.4)	0.4 (0.1 to 1.5)	0.5 (0.2 to 1.4)	0.9 (0.3 to 3.2)	1.9 (0.7 to 5.9)
Man (28) First convulsions^g^	107	55.7 (49.0 to 63.0)	0.4 (0.2 to 0.8)^j^	1.0 (0.4 to 2.3)	2.3 (0.8 to 6.8)	2.3 (1.0 to 5.1)^j^	1.6 (0.7 to 3.5)	0.4 (0.2 to 0.8)^j^
Man (24) Chest pain^c^	172	88.2 (83.6 to 92.8)	0.5 (0.2 to 1.4)	4.2 (0.5 to 33.2)	1.0 (0.3 to 3.8)	2.2 (0.6 to 8.5)	1.0 (0.4 to 2.9)	0.7 (0.2 to 2.3)
Woman (50) Breast lump^c^	192	98.5 (96.4 to 100)	0.7 (0.1 to 9.0)	—^i^	—^i^	0.3 (0.0 to 4.5)	—^﻿i^	2.6 (0.2 to 35.0)
Woman (18) Contraception^f^	169	86.2 (81.1 to 90.8)	0.3 (0.1 to 0.9)^j^	1.2 (0.3 to 4.7)	1.8 (0.4 to 7.5)	10.7 (2.1 to 54.0)^j^	0.8 (0.3 to 2.3)	4.5 (1.6 to 12.3)^j^
Man (32) Sexual problems^d^	142	73.2 (66.0 to 79.4)	1.4 (0.7 to 2.8)	2.5 (0.8 to 7.9)	0.6 (0.2 to 1.8)	1.1 (0.5 to 2.6)	1.4 (0.6 to 3.5)	1.3 (0.6 to 2.7)
Child (13) Physical abuse^h^	73	38.6 (31.7 to 46.0)	0.7 (0.4 to 1.4)	0.9 (0.4 to 2.2)	1.6 (0.6 to 4.2)	1.4 (0.7 to 3.0)	1.5 (0.7 to 3.3)	0.6 (0.3 to 1.2)
Man (45) Anxiety^e^	183	94.8 (91.7 to 97.4)	1.2 (0.3 to 4.6)	0.7 (0.1 to 3.9)	0.9 (0.1 to 8.1)	0.8 (0.2 to 4.0)	0.5 (0.1 to 2.7)	1.1 (0.2 to 5.1)
Relationship problems^g^	77	40.1 (32.8 to 47.4)	0.7 (0.4 to 1.4)	0.8 (0.4 to 2.0)	0.8 (0.3 to 2.3)	1.2 (0.6 to 2.6)	1.4 (0.6 to 3.0)	0.6 (0.3 to 1.2)
Woman (50) Psychosocial problems^e^	182	94.3 (90.7 to 97.4)	0.3 (0.1 to 1.6)	—^i^	0.2 (0.1 to 0.9)^j^	1.4 (0.3 to 7.9)	5.7 (0.6 to 51.1)	0.5 (0.12.5)
Man (52) Alcohol problems^c^	117	60.0 (53.8 to 66.7)	1.1 (0.6 to 2.0)	1.6 (0.7 to 3.8)	0.5 (0.2 to 1.3)	1.1 (0.5 to 2.4)	1.6 (0.73.6)	1.1 (0.52.2)

^a^GPs who answered always or usually. ^b^Missing data = 1. ^c^Missing data = 3. ^d^Missing data = 4. ^e^Missing data = 5. ^f^Missing data = 2. ^g^Missing data = 6. ^h^Missing data = 9. ^i^System missing due to overflow. ^j^ *P*<0.05. ^k^Statistically significant (*P*≤0.002).

Table 3 summarises the patients' answers to three different questions concerning healthcare-seeking behaviour. Almost all patients believed that most people would see their GP for common somatic conditions, such as stomach pain, blood in stools, or children with cough, whereas there was more variation in the patients' answers regarding psychosocial problems such as relationship problems (31.9%) and anxiety (84.5%). Fewer male than female patients expected that patients would seek their GP for anxiety, a cut in need of stiches, help to quit smoking, or sprained ankle. However, more males than females thought it important to see their GP for headache. Compared with responders aged 30–65 years old, younger patients less often believed that patients would see their GP for anxiety or a cut in need of stitches. The oldest group of patients (aged >65 years) were more likely to believe that patients would consult their GP for a sprained ankle or relationship problems. Younger patients found it less important than older patients to see a doctor for several symptoms of possible serious disease. Older patients (aged >65 years) expected to benefit more than the younger patients from a GP visit for stomach problems or nervousness. Almost all patients felt that doctors in general can be trusted.

**Table 3.** Patients’ views on anticipated healthcare-seeking behaviour. Responses from patients (*N* ﻿= 1529, *n* = valid responses) participating in the Norwegian part of the QUALICOPC study, 2012–2013. Results given as valid percentages and ORs with 95% CIs by sex, age, and location

**1. Would most patients see their GP for the following conditions?^ab^**						
			**Sex** **(reference: female)**	**Age groups** **(reference: 30–65 years)**		**Geographical location** **(reference: rural)**
**Condition (valid** **response, *n*)**	**Yes, *n***	**% (95% CI)**	**Male OR (95% CI)**	**Age <30 OR (95% CI)**	**Age >65 OR (95% CI)**	**Urban OR (95% CI)**
Child with severe cough (1372)	1295	94.4 (93.1 to 95.5)	0.6 (0.3 to 0.9)^g^	1.1 (0.5 to 2.3)	0.9 (0.5 to 1.8)	1.3 (0.7 to 2.2
Stomach pain (1411)	1307	92.6 (91.2 to 93.9)	0.8 (0.5 to 1.2)	0.7 (0.4 to 1.2)	1.2 (0.7 to 2.1)	1.1 (0.7 to 1.7)
Deteriorated vision (1356)	927	68.4 (65.9 to 70.8)	1.1 (0.8 to 1.4)	0.8 (0.6 to 1.1)	1.5 (1.1 to 2.1)^g^	0.7 (0.5 to 0.9)^g^
Sprained ankle (1364)	992	67.6 (65.1 to 70.0)	0.7 (0.5 to 0.9)^f^	0.8 (0.6 to 1.2)	1.9 (1.3 to 2.6)^f^	0.8 (0.6 to 1.0)
Cut finger, needing stitches (1391)	1015	73.0 (70.6 to 75.3)	0.6 (0.4 to 0.7)^f^	0.6 (0.4 to 0.8)^f^	1.6 (1.1 to 2.4)^g^	0.6 (0.4 to 0.8)^f^
Wart removal (1324)	1083	81.8 (79.7 to 83.8)	0.7 (0.5 to 1.0)^g^	0.8 (0.5 to 1.2)	0.9 (0.6 to 1.4)	1.2 (0.9 to 1.7)
Blood in stool (1418)	1378	97.2 (96.2 to 98.0)	0.5 (0.3 to 1.0)^g^	0.5 (0.2 to 1.0)^g^	1.5 (0.5 to 3.9)	0.7 (0.3 to 1.6)
Help to quit smoking (1118)	676	60.5 (57.6 to 63.3)	0.6 (0.5 to 0.8)^f^	0.9 (0.6 to 1.2)	0.9 (0.6 to 1.2)	1.0 (0.7 to 1.3)
Sexual problems (1107)	680	61.4 (58.5 to 64.3)	1.3 (1.0 to 1.7)	1.0 (0.7 to 1.4)	0.8 (0.6 to 1.1)	1.0 (0.8 to 1.4)
Domestic violence (1034)	603	58.3 (55.3 to 61.3)	0.9 (0.7 to 1.2)	0.7 (0.5 to 1.0)^g^	1.7 (1.2 to 2.4)^g^	1.0 (0.7 to 1.3)
Anxiety (1284)	1085	84.5 (82.5 to 86.4)	0.4 (0.3 to 0.6)^f^	0.4 (0.3 to 0.6)^f^	1.0 (0.7 to 1.6)	1.0 (0.7 to 1.4)
Relationship problems (1061)	338	31.9 (29.1 to 34.7)	0.8 (0.6 to 1.1)	0.7 (0.5 to 1.1)	1.9 (1.3 to 2.6)^f^	1.0 (0.7 to 1.4)
Routine health cheque (1437)	1356	94.4 (93.1 to 95.5)	0.7 (0.4 to 1.2)	0.5 (0.3 to 0.9)^g^	1.6 (0.8 to 3.4)	1.0 (0.6 to 1.7)
Advice for choosing hospital or specialist (1235)	1061	85.9 (83.9 to 87.8)	1.2 (0.8 to 1.7)	0.6 (0.4 to 0.9)^g^	1.9 (1.1 to 3.2)^g^	0.9 (0.6 to 1.4)
**2. How important would it be for you to see a doctor if you had…?^c^**						
	**Very important**	**%**	**Male OR (95% CI)**	**Age <30 OR (95% CI)**	**Age >65 OR (95% CI)**	**Urban OR (95% CI)**
Weight loss >2 kg in one month (1431)	527	36.8 (34.4 to 39.3)	0.9 (0.7 to 1.2)	0.5 (0.4 to 0.7)^f^	2.3 (1.7 to 3.0)^f^	1.1 (0.8 to 1.4)
Shortness of breath (1429)	792	55.4 (52.8 to 58.0)	1.1 (0.9 to 1.4)	0.6 (0.4 to 0.8)^f^	1.8 (1.3 to 2.4)^f^	1.2 (0.9 to 1.5)
Chest pain when exercising (1424)	1136	79.8 (77.6 to 81.8)	1.0 (0.8 to 1.3)	0.5 (0.3 to 0.6)^f^	1.9 (1.3 to 2.9)^g^	1.0 (0.7 to 1.3)
Headache >1 day (1415)	635	44.9 (42.3 to 47.5)	1.5 (1.2 to 1.9)^f^	0.8 (0.6 to 1.2)	1.7 (1.3 to 2.2)^f^	1.0 (0.8 to 1.3)
Abdominal pains >1 day (1424)	746	52.4 (49.8 to 55.0)	1.3 (1.1 to 1.7)^g^	1.1 (0.8 to 1.5)	1.6 (1.2 to 2.1)^f^	0.9 (0.7 to 1.1)
Loss of consciousness/fainting (1432)	1323	92.4 (90.9 to 93.7)	1.2 (0.8 to 1.8)	0.3 (0.2 to 0.5)^f^	1.4 (0.7 to 2.8)	0.8 (0.5 to 1.3)
Severe worries >1 month (1429)	991	69.3 (66.9 to 71.7)	1.2 (0.9 to 1.5)	1.2 (0.9 to 1.7)	0.8 (0.6 to 1.0)	1.1 (0.8 to 1.4)
**3. Do you expect to benefit from a visit to your GP for...?^d^**						
	**Yes**	**%**	**Male OR (95% CI)**	**Age <30 OR (95% CI)**	**Age >65 OR (95% CI)**	**Urban OR (95% CI)**
Stomach problems (1319)	1189	90.1 (88.5 to 91.7)	1.0 (0.6 to 1.4)	0.7 (0.5 to 1.2)	3.5 (1.7 to 7.4)^f^	1.0 (0.6 to 1.5)
Diarrhoea (1296)	1022	78.9 (76.6 to 81.0)	1.0 (0.8 to 1.4)	0.6 (0.4 to 0.9)^g^	1.9 (1.2 to 2.9)^g^	1.4 (1.0 to 1.9)^g^
Shoulder/neck pain (1295)	1037	80.1 (77.8 to 82.2)	1.2 (0.9 to 1.6)	0.6 (0.4 to 0.9)^g^	1.5 (1.0 to 2.2)	0.8 (0.5 to 1.1)
Headache (1254)	862	68.7 (66.1 to 71.3)	0.8 (0.61.0)	1.0 (0.7 to 1.5)	1.0 (0.7 to 1.4)	1.2 (0.9 to 1.6)
Flu (1301)	857	65.9 (63.3 to 68.4)	0.8 (0.6 to 1.1)	1.1 (0.8 to 1.6)	1.3 (1.0 to 1.8)	1.1 (0.9 to 1.5)
Sore throat (1320)	867	65.7 (63.1 to 68.2)	0.8 (0.6 to 1.0)^g^	0.8 (0.6 to 1.1)	1.2 (0.9 to 1.7)	1.2 (1.0 to 1.6)
Feeling nauseous (1205)	732	60.7 (58.0 to 63.5)	0.8 (0.6 to 1.0)^g^	0.8 (0.6 to 1.2)	1.2 (0.9 to 1.6)	1.4 (1.1 to 1.9)^g^
Feeling tired (1168)	854	73.1 (70.5 to 75.6)	0.6 (0.4 to 0.8)^f^	0.6 (0.4 to 0.9)^g^	1.1 (0.7 to 1.6)	1.3 (1.0 to 1.8)
Feeling nervous (1062)	699	65.8 (62.9 to 68.6)	0.8 (0.6 to 1.0)	0.5 (0.3 to 0.7)^f^	2.0(1.3 to 3.0)^f^	1.0 (0.7 to 1.3)
**4. Do you agree with the following statements?^e^**						
	**Agree**	**%**	**Male OR (95% CI)**	**Age <30 OR (95% CI)**	**Age >65 OR (95% CI)**	**Urban OR (95% CI)**
In general, doctors can be trusted (1458)	1420	97.4 (96.5 to 98.1)	1.4 (0.73.1)	0.8 (0.4 to 2.0)	4.1 (0.9 to 17.3)	2.9 (1.4 to 5.7)^g^
In general, people can be trusted (1396)	1064	76.2 (73.9 to 78.4)	0.9 (0.7 to 1.1)	0.4 (0.3 to 0.6)^f^	1.1 (0.8 to 1.5)	1.2 (0.9 to 1.7)

^a^OR (95% CI), giving the probability of the answer 'Yes' (yes + probably yes); reference is 'No' (no + probably no). ^b'^Don’t know' recoded to missing. ^c^OR gives the probability of 'Important' (extremely + rather important); reference is 'not important' (somewhat + not important). ^d^OR gives the probability for 'Yes', reference is 'No'. ^e^OR gives the probability of 'Agree' (strongly agree + agree), reference is 'Disagree' (disagree+ strongly disagree). ^f^Statistical significance of *P*≤0.002. ^g^ *P*<0.05.

CI = confidence intervals. OR = odds ratio.

**Figure 1:**
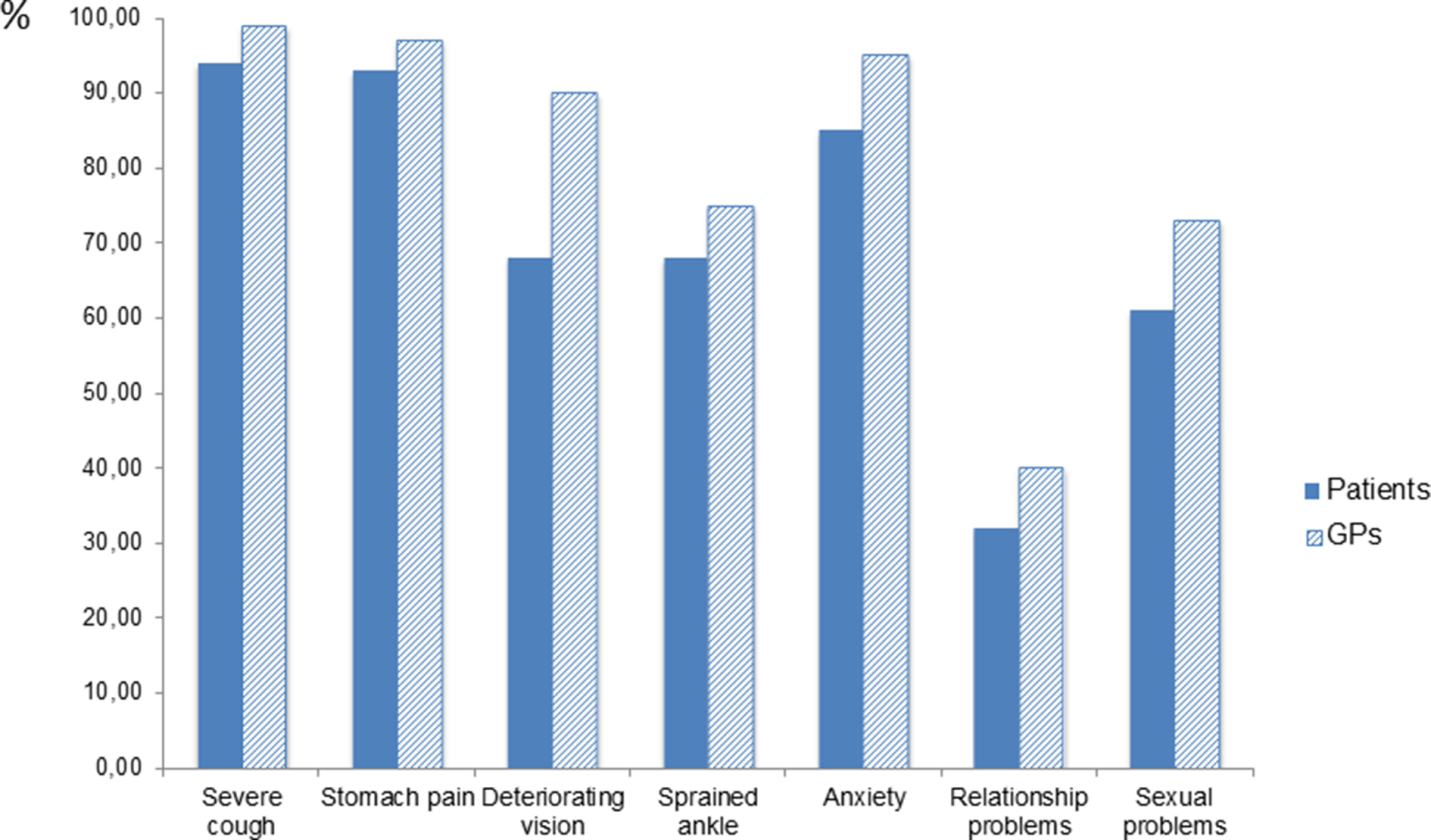
'Will people with the following complaints usually visit a GP?' The columns indicate the percentage of patients that answered 'yes' or 'probably yes', and GPs that answered 'almost always' or 'usually' (details in Tables 2 and 3). For deteriorating vision, anxiety, and sexual problems, the differences were significant when analysed by multiple logistic regression, correcting for GPs’ and patients’ age and sex, location of GP practice, and GPs' list size (Table 4)

For seven health problems or symptoms, there was comparable information from both patients and GPs (Box 1). For all seven items, the GPs were more likely than the patients to believe that people would seek them for the given complaints (Figure 1). In regression analyses, adjusting for the clustered nature of the material and correcting for GPs' and patients' age and sex, size of patient lists, and geographical location of practice, this difference was significant (*P*≤*﻿*0.002) for three of the seven items: deteriorated vision (OR 4.2, 95% CI = 2.5 to 6.9), anxiety (OR 3.0, 95% CI = 1.5 to 6.0), and sexual problems (OR 1.8, 95% CI = 1.3 to 2.6), as shown in Table 4.

**Table 4.** Comparisons of patients’ and their GPs’ expectations regarding healthcare-seeking behaviour. Multiple logistic regression (GEE), corrected for patients’ age and sex, GPs’ age and sex, size of patient lists, and geographical location of practice. OR indicates the probability of the GPs answering Yes, with patients as reference group

Will people with the following complaints usually visit their GP?	GPs (reference: patients)		
	**OR**	**95% CI**	***P* value**
Severe cough	3.4	1.1 to 10.5	0.04
Abdominal pain	2.7	1.2 to 6.5	0.02
Deteriorating vision	4.2^a^	2.5 to 6.9	<0.001
Sprained ankle	1.4	1.0 to 2.0	0.07
Anxiety	3.0^a^	1.5 to 6.0	0.002
Relationship problems	1.5	1.1 to 2.1	0.02
Sexual problems	1.8^a^	1.3 to 2.6	0.001

^a^Indicates significant differences, *P*≤0.002. CI = confidence intervals. GEE = generalised estimating equation. OR = odds ratio.

## Discussion

### Summary

Norwegian GPs seem to overestimate how often patients would visit them for common health problems. This applies in particular for psychosocial problems.

Male patients were less prone to believe that most people will visit a GP for some common conditions. Older patients found it more important than younger and middle-aged patients to see a doctor, and had higher expectations of benefitting from a GP visit.

### Strengths and weaknesses

To the authors' knowledge, existing research on patient expectations has not investigated differences between GPs’ and patients’ expectations regarding which problems patients will seek their GP for.^7,9^ This study therefore provides new knowledge within the field of patient–doctor interaction. This study allows linking of information from patients with information from their GPs. Using a GEE logistic regression model, the authors have adjusted for the patient expectations stemming from variation at the GP-level. GPs and patients were recruited from the whole country, and their age and sex distributions are comparable to the Norwegian averages.^20,21^

Patients were recruited in the GPs’ waiting room, meaning that only patients who had already decided to see a GP were included. Thus, persons with low expectations to benefit from a GP visit were less likely to be included in the study. This may have caused an underestimation of the differences in expectation.

The questionnaires were originally designed for a large international study, and were, among other things, designed to compare the results with a previous study.^22,23^ The phrasing of the questions is slightly different in the GP questionnaire than in the patient questionnaire (Box 1), and this may theoretically have caused an overestimation of the differences. Furthermore, the selection of health problems were decided by the international QUALICOPC consortium and have not been adapted to a Norwegian setting specifically.

### Comparison with existing literature

Literature concerning medical services offered by GPs often focuses on the content of the consultation.^24–28^ However, patients’ thoughts about what kind of problems their GP can assist with are less well described. This study adds new knowledge to this field. Some of the findings seem surprising: <40% of the patients considered it very important to see a doctor if they involuntarily lose 2 kilograms in a month, although unintended weight loss is considered an alarm symptom for possible malignant disease.^29,30^ Only 60% of the patients believed that most patients would see a GP for help to quit smoking. This is in contrast to both public awareness campaigns and extensive research documenting GPs' potentially important role in smoking cessation.^31^ Further research with qualitative methodology may explore possible explanations for these observations.

Several associations were found between patients’ sex or age, and their expectations. When asked whether most patients would see a GP for the selected diagnosis, there was a tendency that male patients less often answered Yes. This is in accordance with the established knowledge that women see their GP more often than men.^4,6,32^

The youngest patients were less likely than middle-aged patients to believe that most patients would see a GP for several of the listed conditions. This could be due to a generational change in self-management of health problems. Younger patients may also be more likely to seek help through new tools such as social media.

Older patients found it more important than younger patients to see a doctor in presence of medical symptoms. This result mirrors the 'pre-test probability' for significant disease that increases with age for a given symptom.

GPs seemed to overestimate to what degree their patients will consult them. With a significance level of *P*≤0.002, only three of the seven items reached significance, but *P* value was <0.05 for all items except one. The authors interpret this as a probable general tendency for GPs to overestimate their patients’ expectations. The difference seen for deteriorating vision is most likely due to easily accessible optometrists in Norway, who can also refer to ophthalmologists if needed. As for anxiety and sexual problems, some people may not be aware that GPs can assist with this kind of problems. It is also possible that anticipated social stigma or embarrassment is a reason for lower patient expectations.

The patients may have considered the illness behaviour of the estimated 75% of the population that report any symptom or illness per month, while the GPs may have considered the smaller part of the population that already have decided that they need professional help, as described by White.^15^ The authors still believe that the observed difference may represent a real divergence in expectations between GPs and patients. For the non-somatic items, there were between 16–31% missing patient answers, possibly reflecting patients’ uncertainty regarding whether their GP can offer help. Therefore, the actual divergence in expectations may be larger than shown.

The authors have not been able to identify other studies that directly compare patients’ and GPs’ attitudes in a similar way. A recent study investigated patients' propensity to seek health care in different healthcare systems.^16^ The organisation of primary care, as well as patients’ perceived communication with their GP, was highly correlated with patients’ decision to seek health care, but the authors did not look into GPs’ attitudes. A recent Swiss study found that GPs underestimate the satisfaction of their patients.^17^ A Dutch study from 1999 found that GPs working within a referral system, like in Norway, saw themselves as the likely first point of healthcare contact for patients with psychosocial problems, but patients’ attitudes were not reported.^23^

### Implications for practice

Both age and sex influence patients’ expectations to what GPs can help them with. Older patients have higher expectations of benefitting from a GP visit and find it more important than younger patients to see a GP in the presence of several health complaints. The results suggest that Norwegian GPs overestimate to what degree their patients will see them for a variety of common medical problems, in particular psychosocial issues. Patient-centred health services necessitate knowledge concerning which types of problems patients are likely to consult for, and patients must be informed about the services offered by GPs. If the observed differences represent an actual divergence in expectations between GPs and patients, it should have implications for measures taken to contribute to a more rational and cost-efficient use of healthcare services.
